# Heterogeneity of Latency Establishment in the Different Human CD4^+^ T Cell Subsets Stimulated with IL-15

**DOI:** 10.1128/jvi.00379-22

**Published:** 2022-05-02

**Authors:** Giacomo M. Butta, Giorgio Bozzi, Greta Gallo, Gaia Copaloni, Chiara Cordiglieri, Mariacristina Crosti, Marilena Mancino, Daniele Prati, Viviana Simon, Andrea Gori, Alessandra Bandera, Raffaele De Francesco, Lara Manganaro

**Affiliations:** a INGM-Istituto Nazionale di Genetica Molecolare, Virology, Milan, Italy; b Università degli Studi di Milano, Department of Pharmacological and Biomolecular Sciences, Milan, Italy; c Fondazione IRCCS Ca' Granda Ospedale Maggiore Policlinico, Infectious Diseases Unit, Department of Internal Medicine, Milan, Italy; d Department of Transfusion Medicine and Hematology, Fondazione IRCCS Cà Granda, Ospedale Maggiore Policlinico, Milan, Italy; e Department of Microbiology, Icahn School of Medicine at Mount Sinai, New York, New York, USA; f Global Health Emerging Pathogens Institute, Icahn School of Medicine at Mount Sinai, New York, New York, USA; g Division of Infectious Diseases, Department of Medicine, Icahn School of Medicine at Mount Sinai, New York, New York, USA; h Università Degli Studi di Milano, Department of Pathophysiology and Transplantation, Centre for Multidisciplinary Research in Health Science, (MACH), Milan, Italy; Emory University

**Keywords:** CD4 T subsets, HIV, IL-15, latency

## Abstract

HIV integrates into the host genome, creating a viral reservoir of latently infected cells that persists despite effective antiretroviral treatment. CD4-positive (CD4^+^) T cells are the main contributors to the HIV reservoir. CD4^+^ T cells are a heterogeneous population, and the mechanisms of latency establishment in the different subsets, as well as their contribution to the reservoir, are still unclear. In this study, we analyzed HIV latency establishment in different CD4^+^ T cell subsets stimulated with interleukin 15 (IL-15), a cytokine that increases both susceptibility to infection and reactivation from latency. Using a dual-reporter virus that allows discrimination between latent and productive infection at the single-cell level, we found that IL-15-treated primary human CD4^+^ T naive and CD4^+^ T stem cell memory (T_SCM_) cells are less susceptible to HIV infection than CD4^+^ central memory (T_CM_), effector memory (T_EM_), and transitional memory (T_TM_) cells but are also more likely to harbor transcriptionally silent provirus. The propensity of these subsets to harbor latent provirus compared to the more differentiated memory subsets was independent of differential expression of pTEFb components. Microscopy analysis of NF-κB suggested that CD4^+^ T naive cells express smaller amounts of nuclear NF-κB than the other subsets, partially explaining the inefficient long terminal repeat (LTR)-driven transcription. On the other hand, CD4^+^ T_SCM_ cells display similar levels of nuclear NF-κB to CD4^+^ T_CM_, CD4^+^ T_EM_, and CD4^+^ T_TM_ cells, indicating the availability of transcription initiation and elongation factors is not solely responsible for the inefficient HIV gene expression in the CD4^+^ T_SCM_ subset.

**IMPORTANCE** The formation of a latent reservoir is the main barrier to HIV cure. Here, we investigated how HIV latency is established in different CD4^+^ T cell subsets in the presence of IL-15, a cytokine that has been shown to efficiently induce latency reversal. We observed that, even in the presence of IL-15, the less differentiated subsets display lower levels of productive HIV infection than the more differentiated subsets. These differences were not related to different expression of pTEFb, and modest differences in NF-κB were observed for CD4^+^ T naive cells only, implying the involvement of other mechanisms. Understanding the molecular basis of latency establishment in different CD4^+^ T cell subsets might be important for tailoring specific strategies to reactivate HIV transcription in all the CD4^+^ T subsets that compose the latent reservoir.

## INTRODUCTION

Highly active antiretroviral therapy (HAART) effectively suppresses HIV replication but fails to eradicate the virus from the host. The main obstacle to HIV eradication is the persistence of a latent reservoir that is invisible to both humoral and cell-mediated immunity (1–3).

The latent reservoir is formed early upon infection in both CD4-positive (CD4^+^) T lymphocytes and myeloid cells, but resting memory CD4^+^ T cells harbor the majority of proviral DNA (3–6). The persistence of the HIV reservoir relies on the long half-life of resting memory CD4^+^ T cells and their proliferation (7, 8). Indeed, CD4^+^ T cells carrying a competent but latent provirus can expand clonally without viral reactivation (9–16). Several studies have been performed to characterize cells that comprise the HIV latent reservoir (17–25). CD4^+^ T lymphocytes are a heterogeneous population, characterized by subsets that differ in phenotype, function, and maturation level (26). The common way to define these subsets is based on their level of differentiation, from undifferentiated cells that have not encountered the antigen to the most differentiated subset. These different subsets include naive, T stem cell memory (T_SCM_), central memory (T_CM_), transitional memory (T_TM_), and effector memory (T_EM_) cells (26). Naive T cells have not encountered their specific antigen yet and undergo differentiation into memory cells once exposed to such specific antigens. T_CM_ cells are long-lived cells that are responsible for the recall responses, while T_EM_ cells migrate to inflammatory sites and mediate the rapid response. T_TM_ cells are in a transient state between T_CM_ and T_EM_, while T_SCM_ cells are long-lived, minimally differentiated memory cells with a high capacity for self-renewal (26–28).

Susceptibility to infection, latency establishment, and reactivation differ in the distinct CD4^+^ T cell subsets. Naive CD4^+^ T cells are the most resistant to HIV infection, possibly due to poor expression of CCR5, low F-actin density, low levels of the phosphorylated form of SAMHD1, or general differences in cellular metabolism, while T_EM_ and T_TM_ cells are the most susceptible (29–35). CD4^+^ T_CM_ cells have been reported to be the major contributor to the general latent reservoir in several studies (16, 22, 23). Recently, new evidence suggests that T_EM_ cells are the largest contributors to the inducible reservoir and that differentiation of memory subsets into an effector memory phenotype enhances the latency reversal with specific γ-chain and dendritic cell (DC)-specific cytokine stimulation (36, 37). It has also been shown that HIV preferentially establishes a latent infection in a specific CD4^+^ T cell population identified as an effector of memory transitioning population and that HIV silencing correlates with downregulation of NF-κB (38).

Latency reversing agents (LRA) have been tested for their ability to purge the latent reservoir with very mixed, mostly less than optimal, results (39–42).

The reactivation of HIV latent provirus in the different CD4^+^ T cell subsets has been a subject of intense research in the past years (37, 41, 43–45).

Indeed, the response to LRA treatment in the different CD4^+^ T cell subsets has been shown to be heterogeneous, while after stimulation with phytohemagglutinin (PHA), all memory T cell subsets displayed similar levels of HIV transcription induction (37, 41, 43, 44).

This heterogeneity highlights the critical need to better define whether LRA compounds are effective in reactivating HIV transcription in all the different T cell subsets.

It has been recently shown that activation of the interleukin 15 (IL-15) pathway through IL-15 superagonist N-803 coupled with CD8^+^ T cells depletion is effective in inducing viral transcription *ex vivo* and in animal models (46). However, IL-15 is also upregulated during acute HIV/simian immunodeficiency virus (SIV) infection when the reservoir is established (47, 48). Moreover, it also increases susceptibility to infection of CD4^+^ T cells (35, 47). The impact of IL-15 on HIV latency establishment in different subsets is still unknown.

To study the potential role of IL-15 in HIV latency establishment in different CD4^+^ T cell subsets, we took advantage of the second-generation dual-reporter virus HIV-GKO that distinguishes latently and productively infected cells at the single-cell level (49, 50). We observed that naive and T_SCM_ cells are more prone to latency than the other subsets. Analysis of three important components for HIV transcription, cyclin T1, CDK9, and NF-κB, revealed that expression of pTEFb components was comparable among the CD4^+^ T subsets, while nuclear localization of NF-κB was diminished only in naive T cells (51–53). These data suggest that after IL-15 treatment, *in vitro* HIV latency establishment is heterogeneous and that other cellular components, rather than availability of transcription factors, are important for HIV latency establishment, especially in T_SCM_, a long-lived population that plays a critical role in both latency maintenance and HIV/SIV pathogenesis (54–56).

## RESULTS

### IL-15 increases the susceptibility to HIV infection and LTR-driven transcription in CD4^+^ T cells.

We used HIV-GKO, a second-generation dual-fluorescent virus that carries green fluorescent protein (csGFP) under the control of the HIV LTR promoter and Kusabira Orange (mKO2) under the control of the cellular promoter EF1α (49, 50). Single-cycle infection with the reporter virus supports studies of latency establishment in human primary CD4^+^ T cells by providing accurate quantification of both latent and productive infections. HIV-GKO productively infected cells express both csGFP and mKO2, while latently infected cells only express mKO2.

**FIG 1:**
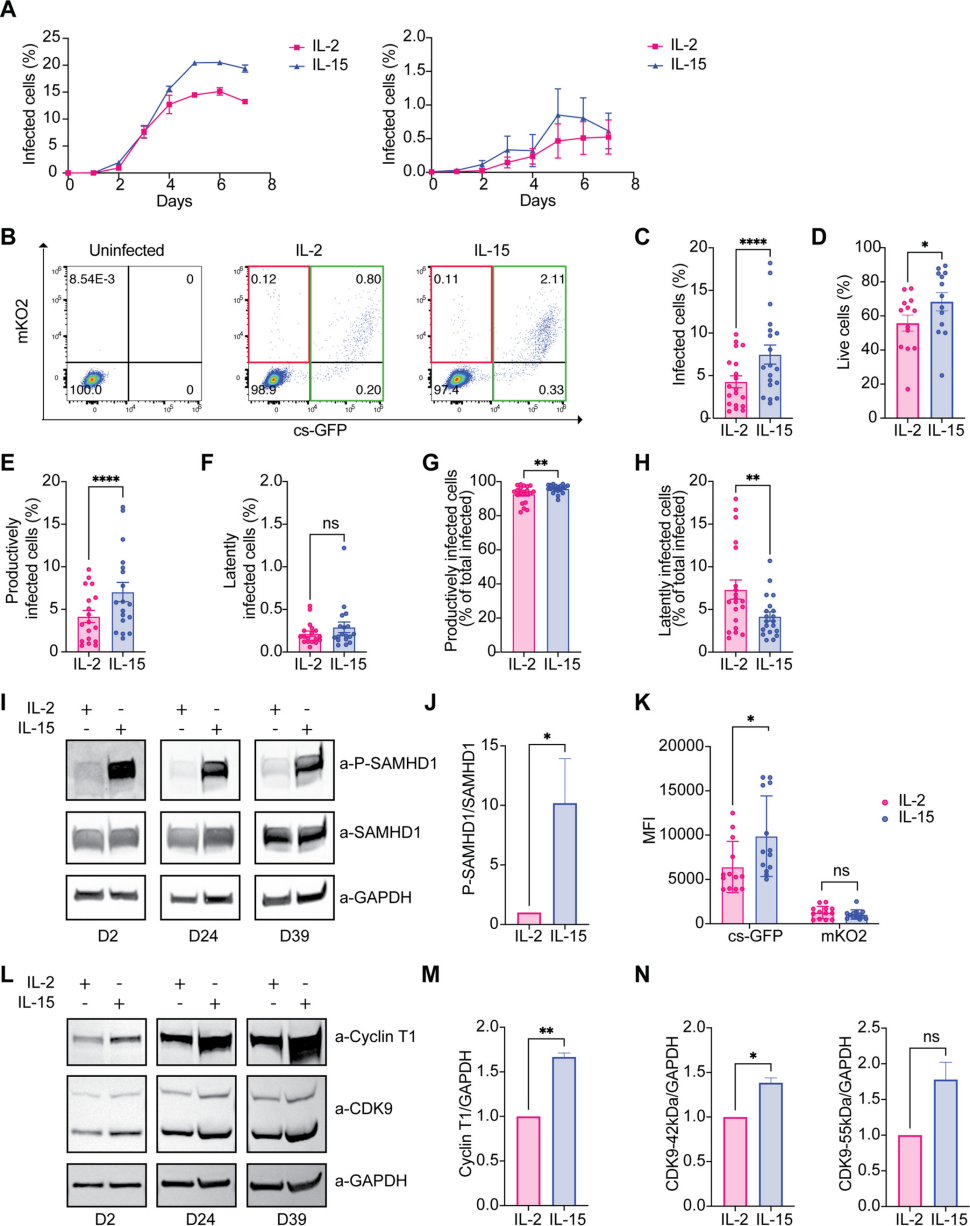
IL-15 increases the susceptibility to R5/X4 HIV infection and LTR-driven transcription in CD4^+^ T cells. (A) R5/X4 HIV-GKO infection time course at high multiplicity of infection (MOI) (left) and low MOI (right). CD4^+^ cells were infected with R5/X4 HIV-GKO, and the percentage of infected cells was analyzed by flow cytometry (*n* = 3). (B) Representative FACS plots showing the gating strategy to discriminate between latently and productively infected cells. CD4^+^ T latently infected population was defined as mKO2^+^/csGFP^−^; productive population was defined as csGFP^+^/mKO2^−^ and csGFP^+^/mKO2^+^. (C) Bar graph showing the percentage of total infected cells (mKO2^+^/csGFP^−^, csGFP^+^/mKO2^+^, and csGFP^+^/mKO2^−^) after IL-2 or IL-15 stimulation. (D) Percentage of live cells in the two conditions is shown. (E and F) Bar graphs showing the percentage of productively infected cells (csGFP^+^/mKO2^−^ and csGFP^+^/mKO2^+^) (E) and latently infected cells (mKO2^+^/csGFP^−^) (F) with respect to total CD4^+^ T cells. (G and H) Bar graphs showing the percentage of productively infected cells (G) and latently infected cells (H) with respect to the total infected cells. (I) Cell lysates from total CD4^+^ T cells from 3 donors were analyzed by immunoblotting for P-SAMHD1, SAMHD1, and GAPDH after 3 days of stimulation with equimolar concentrations of IL-2 and IL-15. (J) Densitometric quantification of P-SAMHD1 levels with respect to total SAMHD1 after IL-2 or IL-15 stimulation in CD4^+^ T cells (*n* = 3). (K) Mean fluorescence intensity (MFI) of csGFP and mKO2 in CD4^+^ T cells infected with R5/X4 HIV-GKO after IL-2 or IL-15 stimulation. (L) Cell lysates from 3 different donors were analyzed by immunoblotting for cyclin T1 and CDK9 expression after 8 days of stimulation with equimolar concentrations of IL-2 and IL-15. (M and N) Densitometric quantification of cyclin T1, CDK9 42 kDa, and CDK9 55 kDa in respect to housekeeping GAPDH, respectively, after IL-2 or IL-15 stimulation (*n* = 3). Significance was determined by Wilcoxon matched-pairs rank test. *, *P* ≤ 0.05; **, *P* ≤ 0.01; ***, *P* ≤ 0.001; ****, *P* ≤ 0.0001.

Since HIV-GKO lacks a functional envelope, we generated a single-cycle virus by pseudotyping it with a dual-tropic (R5/X4) HIV-1 subtype B envelope (R5/X4 HIV-GKO) (57). First, we monitored total infection with R5/X4 HIV-GKO (csGFP^+^/mKO2^+^, csGFP^+^/mKO2^−^, and mKO2^+^/csGFP^−^) over 7 days, in primary CD4^+^ T cells stimulated with IL-2 or IL-15 at equimolar concentration. We infected CD4^+^ T cells with a high dose of R5/X4 HIV-GKO (500 ng p24 per million cells) (Fig. 1A, left) and a low dose (50 ng p24 per million cells) (Fig. 1A, right). The infection with R5/X4 HIV-GKO peaked between day 5 and day 6 regardless of the viral input. We observed that the overall rate of infection was increased in cells stimulated with IL-15 compared to cells stimulated with IL-2 (Fig. 1A).

For the next experiments, we decided to use an intermediate dose of viral input (300 ng/million cells) and to analyze cells 5 to 6 days postinfection. Expression of csGFP and mKO2 was monitored by flow cytometry; csGFP^+^/mKO2^+^ and csGFP^+^/mKO2^−^ were defined as productively infected cells, while mKO2^+^/csGFP^−^ were defined as latently infected cells as previously reported (Fig. 1B) (49).

We observed that R5/X4 HIV-GKO total infection (productive and latent), as well as cell viability, were higher in CD4^+^ T cells stimulated with IL-15 compared to IL-2 stimulated (Fig. 1C and D).

We next analyzed differences in productively and latently infected cells in IL-15 and IL-2 conditions. The percentage of productively infected cells was increased in CD4^+^ T cells stimulated with IL-15 compared to IL-2 stimulated, while the percentage of latently infected cells remained the same (Fig. 1E and F). We then calculated the percentage of productively and latently infected cells within the total amount of infected cells. As shown in Fig. 1G, the majority of infected cells are productive, while only a small percentage is latent (Fig. 1H). Of note, we observed that in IL-2-stimulated CD4^+^ T lymphocytes, the proportion of productively infected cells was lower, while the proportion of latently infected cells was higher than the IL-15 condition (Fig. 1G and H).

The increased infection in CD4^+^ T cells stimulated with IL-15 was associated with an almost 10-fold increase in the phosphorylation of SAMHD1 on threonine 592 that abrogates its restriction activity (Fig. 1I and J) (35, 58, 59).

The increase in productive infection suggested that IL-15 could also have a role in HIV transcription. Indeed, the mean fluorescence intensity (MFI) of csGFP, which is under the control of the LTR promoter, was increased in infected cells stimulated with IL-15, while mKO2 MFI remained stable, suggesting that IL-15 does not only relieve an early block in HIV infection but also increases LTR-driven transcription (Fig. 1K).

We investigated next whether IL-15 stimulation could modulate the expression of pTEFb complex, an important player in LTR-driven transcription (51, 53). We stimulated CD4^+^ T cells from three different donors with equimolar concentrations of IL-2 and IL-15 and analyzed the expression of CDK9 and cyclin T1 after 8 to 9 days of stimulation, a time when latent and productive infection with HIV-GKO was measured (Fig. 1L). We observed an average 50% increase in cyclin T1 and both isoforms of CDK9 in CD4^+^ T cells stimulated with IL-15 compared to IL-2 (Fig. 1M and N).

Taken together, these data suggest that IL-15, in addition to inducing SAMHD1 phosphorylation, also upregulates expression of pTEFb, thus increasing both susceptibility to HIV infection as well as viral transcription in total CD4^+^ T lymphocytes.

### R5/X4 HIV-GKO infection does not alter CD4^+^ T cell subset distribution.

**FIG 2:**
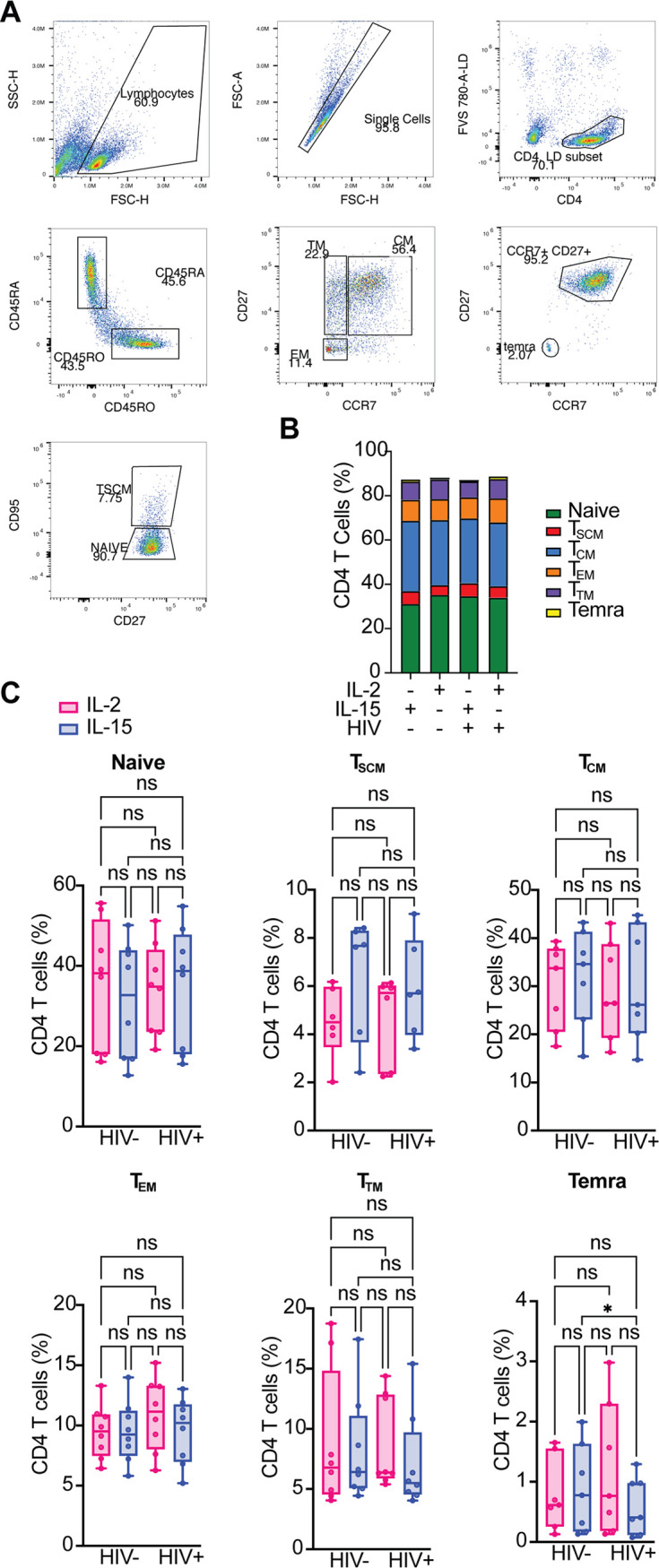
R5/X4 HIV-GKO infection does not alter CD4^+^ T cell subset distribution. (A) Representative gating strategy to discriminate among the different CD4^+^ T cell subsets is shown. Naive cells were identified as CD45RO^−^ CD45RA^+^ CD27^+^ CCR7^+^ CD95^−^, T_SCM_ cells were defined as CD45RO^−^, CD45RA^+^ CD27^+^ CCR7^+^ CD95^+^, Temra cells were defined as CD45RO^−^ CD45RA^+^ CD27^−^ CCR7^−^, T_CM_ cells were defined as CD45RO^+^ CD45RA^−^ CD27^+^ CCR7^+^, T_TM_ cells were defined as CD45RO^+^ CD45RA^−^ CD27^+^ CCR7^−^, and T_EM_ cells were defined as CD45RO^+^ CD45RA^−^ CD27^−^ CCR7^−^. (B) Stacked bar chart shows the average distribution of the different CD4^+^ T cell subsets in the indicated conditions. (C) Bar graphs showing the percentage of each CD4^+^ T cell subset in the different conditions analyzed. Significance was determined by Friedman test. ns, *P* > 0.05; *, *P* ≤ 0.05.

Next, we analyzed the distribution of six CD4^+^ T cell subsets (naive, T_SCM_, T_CM_, T_EM_, T_TM_, and Temra) stimulated with IL-15 or IL-2 in the presence or the absence of R5/X4 HIV-GKO infection. CD4^+^ T cell subsets were classified by the expression of specific membrane markers. Specifically, we used differential expression of CD45RA, CD45RO, CD27, CCR7, and CD95 to discriminate among naive CD4^+^ T cells, Temra, T_CM_, T_EM_, T_TM_, and T_SCM_ cells (Fig. 2A) (26, 60). The average distribution of all CD4^+^ T cell subsets was similar in the different conditions, with a decrease in Temra cells in the IL-15 condition after infection (Fig. 2B and C). An increase in T_SCM_ cells was observed in CD4^+^ T cells stimulated with IL-15, but this difference did not reach statistical significance. Overall, these data indicate that infection with R5/X4 HIV-GKO in the presence of IL-15 or IL-2 does not change the distribution of CD4^+^ T lymphocytes.

### Productive infection is more frequent in T_CM_, T_EM_, and T_TM_ cells.

We next analyzed the establishment of latency in the different CD4^+^ T cell memory subsets and naive cells. Temra cells were left out from this analysis due to their low number, short life span, and high variability among donors. Primary CD4^+^ T cells were stimulated for 72 h with IL-15 or IL-2 and infected with R5/X4 HIV-GKO. Five days after infection, CD4^+^ T cells were stained with the previously described cell surface markers to identify infection in the different subsets by flow cytometry. To better visualize the distribution of productively and latently infected cells, we employed t-distributed stochastic neighbor embedding (t-SNE) plots, which show how phenotypically similar cells cluster together, while phenotypically diverse cells cluster far away (61).

**FIG 3:**
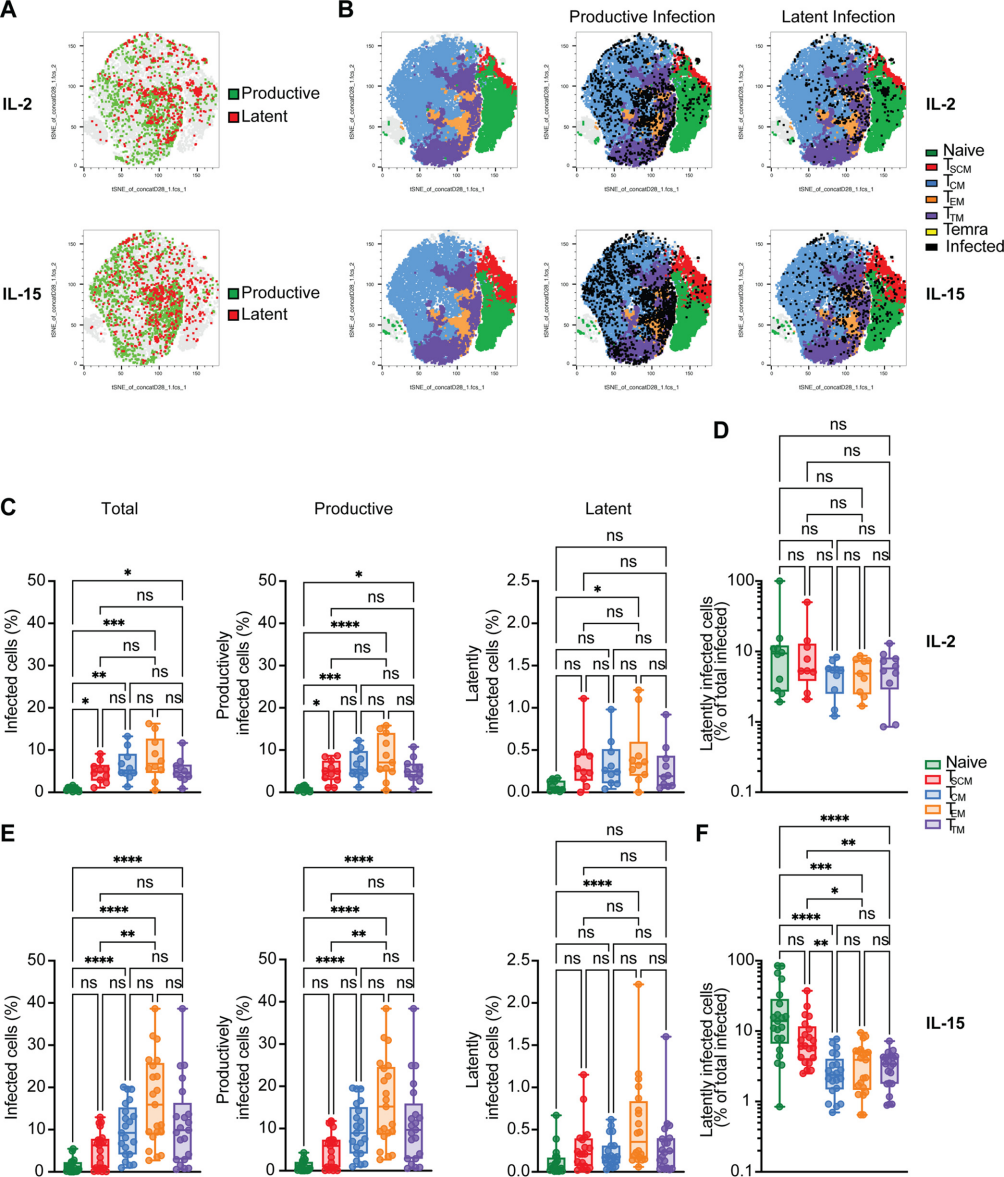
Productive infection is more frequent in T_CM_, T_EM_, and T_TM_ cells. (A) Representative t-SNE plots showing the distribution of latently (in red) and productively infected cells (in green) within total CD4^+^ T cells in the two different conditions analyzed. (B) Representative t-SNE plots showing the distribution of the different CD4^+^ T cell subsets (left), the distribution of productively infected cells (middle), and the distribution of latently infected cells (right) within the different CD4^+^ T cell subsets in IL-2 condition (top panels) or IL-15 condition (bottom panels). (C) Box plots displaying the percentage of total infected cells (left), productively infected cells (middle), and latently infected cells (right) in the different CD4^+^ T cell subsets after IL-2 stimulation. (D) Box plot showing the percentage of latently infected cells with respect to total infection in each CD4^+^ T cell subset in IL-2 condition. (E) Box plots showing the percentage of total infected cells (left), productively infected cells (middle), and latently infected cells (right) in the CD4^+^ T cell subsets in IL-15 condition. (F) Box plot showing the percentage of latently infected cells with respect to total infection in each CD4^+^ T cell subset after IL-15 stimulation. Significance was determined by Kruskal-Wallis test. *, *P* ≤ 0.05; **, *P* ≤ 0.01; ***, *P* ≤ 0.001; ****, *P* ≤ 0.0001; ns, not significant.

The representative t-SNE plot in Fig. 3A shows the distribution of productively infected cells (in green) and latently infected cells (in red) in IL-15- and IL-2-stimulated CD4^+^ T cells. We observed that productively infected cells clustered together, suggesting a similarity in the immunophenotype of the infected cells. On the other hand, latently infected cells were more scattered throughout the population. To further investigate the different distribution of infected cells within each T cell subset, we included the phenotypic markers in our t-SNE analysis and overlaid both the productive and the latent infection (Fig. 3B). The majority of productively infected cells were clustered with T_CM_ (light blue cluster), T_EM_ (orange cluster), and T_TM_ (purple cluster) (Fig. 3B, middle, “productive infection”). Conversely, latently infected cells had a more heterogeneous distribution (Fig. 3B, right, “latent infection”).

To analyze latency establishment more quantitatively, we determined the outcome of infection in each subset in IL-2 or IL-15 conditions. First, we determined the levels of total, productive, and latent infection in IL-2-stimulated cells. As previously observed, naive T cells were the least susceptible to HIV infection, with an average of 0.8% infected cells, while T_EM_ cells were the most susceptible, with an average of 8% (Fig. 3C, left). A similar distribution was observed for productive infection only (Fig. 3C, middle), while the percentage of latently infected cells was similar in all the memory subsets (Fig. 3C, right). We then calculated the percentage of latently infected cells within the total infected cells pool in each subset. Naive and T_SCM_ cells are slightly more prone to harbor a latent infection than the other memory subsets (Fig. 3D).

Also after IL-15 stimulation, naive CD4^+^ T cells were the most resistant to HIV infection, with an average infection of 1.2%, while T_EM_ cells were the most susceptible, with an average infection of 16.8% (31). Average levels of infection in T_CM_ and T_TM_ cells were 9.8% and 11.5%, respectively, while T_SCM_ cells were the least permissive of the memory subsets, with an average of 5.2% (Fig. 3E, left). We then determined the percentage of productively and latently infected cells in each subset. As for the IL-2 condition, the distribution of productive infection was similar to the total infection (Fig. 3E, middle). In contrast to total infection, the differences in latent infection were less pronounced in the different CD4^+^ T cell subsets. The subset that harbored the majority of latently infected cells was the T_EM_ subset, with an average of 0.5%. The average percentage of latently infected cells in the naive subset was 0.12% and in T_SCM_ was 0.27%; in T_CM_, we observed an average of 0.22% of latently infected cells, while in T_TM_ cell, it was 0.3% (Fig. 3E, right).

We next determined the percentage of latently infected cells with respect to total infection in each subset for the IL-15 condition. We observed that the relative percentage of latently infected cells was higher in naive and T_SCM_ cells than T_CM_, T_EM_, and T_TM_ cells. Specifically, the average percentage of latently infected cells was 23.4% in naive cells, 9.3% in T_SCM_ cells, 2.9% in T_CM_ cells, 3.7% in T_EM_ cells, and 3.2% in T_TM_ cells (Fig. 3F).

Taken together, these data suggest that, in addition to the overall low susceptibility to HIV infection of naive and T_SCM_ cells, once HIV infects these subsets, its transcription is more likely to be silenced than the more differentiated CD4^+^ T subsets.

### IL-15 stimulation increases HIV infection in all the memory subsets.

**FIG 4:**
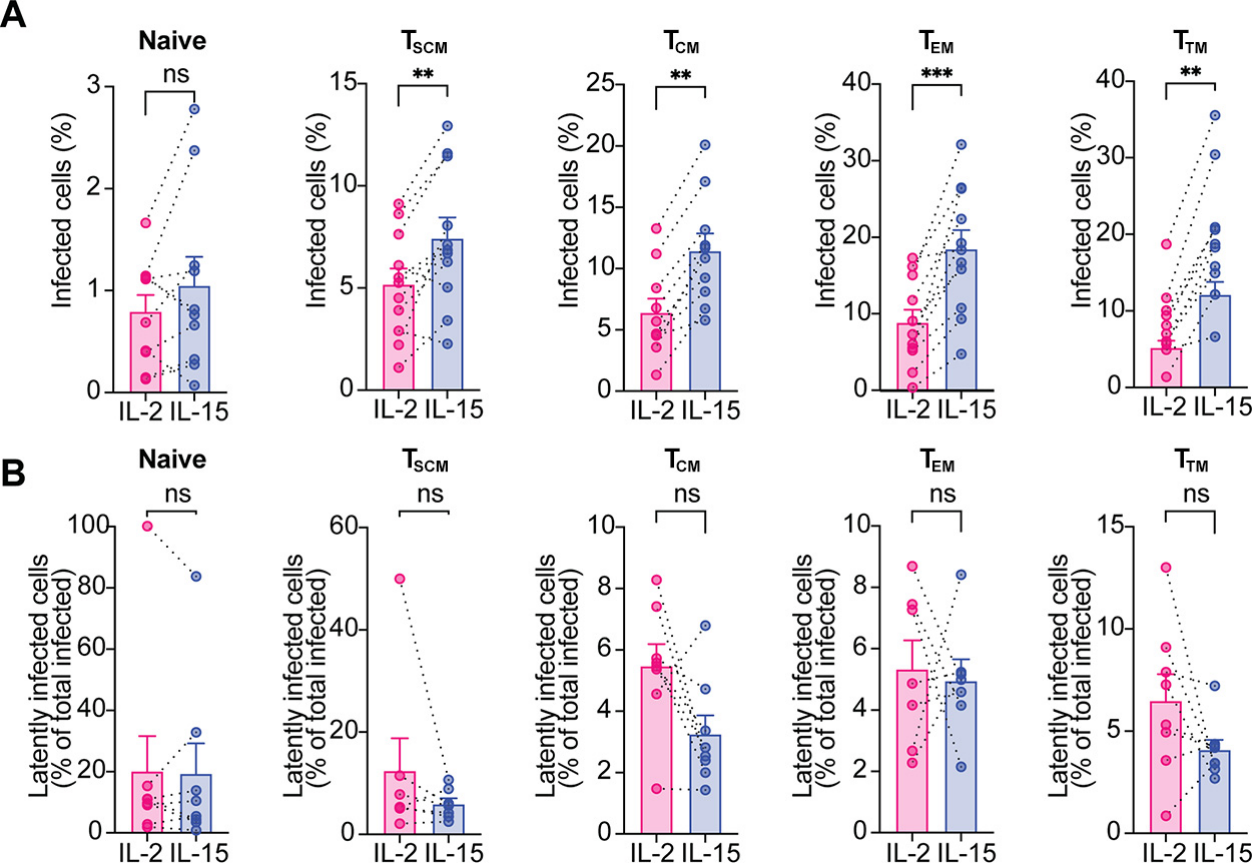
IL-15 stimulation increases HIV infection in all the memory subsets. (A) Bar graphs showing the percentage of total infected cells, in the indicated conditions, for each CD4^+^ T cell subset. (B) Percentage of latently infected cells with respect to total infection, in IL-2 or IL-15 stimulation, for each subset. Significance was determined by Wilcoxon signed-rank test. *, *P* ≤ 0.05; **, *P* ≤ 0.01; ***, *P* ≤ 0.001.

We next compared the overall infection and latency establishment under IL-2 or IL-15 stimulation for all the CD4^+^ T cells subsets. IL-15 significantly increases total infection in all the memory subsets (Fig. 4A) but not in the naive T cells, possibly due to the lower expression of the IL-15 receptor in these cells than in the memory subsets (62). Furthermore, IL-15 had a negative impact on latency establishment in T_SCM_, T_CM_, and T_TM_ cells, although it never reached statistical significance (Fig. 4B).

### Infection with CCR5 tropic GKO is enhanced in the presence of IL-15.

**FIG 5:**
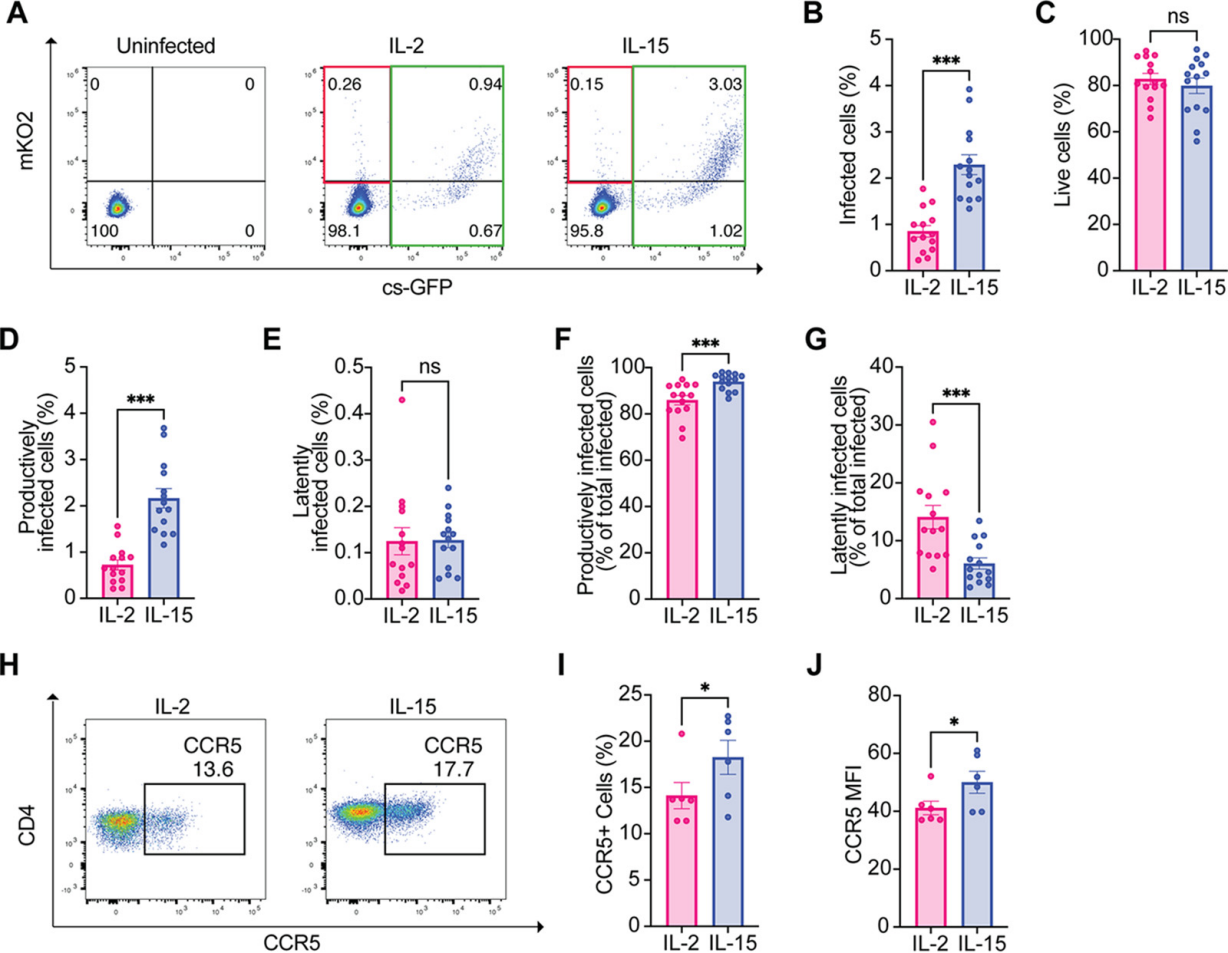
Infection with CCR5 tropic GKO is enhanced in the presence of IL-15. (A) Representative FACS plots showing the gating strategy to discriminate between latently and productively infected cells using R5 HIV-GKO. (B) Bar graph showing the percentage of total infected cells in IL-2- and IL-15-stimulated CD4^+^ T lymphocytes. (C) Graph shows the percentage of live cells in the two conditions. (D and E) Bar graphs showing the percentage of productively infected cells (D) and latently infected cells (E) with respect to total CD4^+^ T cells. (F and G) Bar graphs showing the percentage of productively infected cells (F) and latently infected cells (G) with respect to the total infected cells in the two conditions. (H) Representative FACS plots showing CCR5 expression in CD4^+^ T lymphocytes after 3 days of stimulation with IL-2 or IL-15. (I) Bar graph showing the percentage of CCR5^+^ CD4^+^ cells in the indicated conditions. (J) Mean fluorescence intensity (MFI) of CCR5 in IL-2 and IL-15 stimulation. Significance was determined by Wilcoxon signed-rank test. *, *P* ≤ 0.05; **, *P* ≤ 0.01; ***, *P* ≤ 0.001.

The latent reservoir is established early upon infection, and the viruses associated with acute infection are mainly CCR5 tropic (4, 63–65). We then investigated whether IL-15 stimulation has an impact on infection and latency establishment of HIV-GKO bearing a CCR5-tropic envelope (R5 HIV-GKO) (Fig. 5A). IL-15 increases R5 HIV-GKO total infection, with no impact on cell viability (Fig. 5B and C).

Similar to what we observed with the X4/R5 HIV-GKO, IL-15 increased the percentage of productively R5 HIV-GKO-infected cells but had no impact on the percentage of latently infected cells compared to IL-2 (Fig. 5D and E). When we calculated the percentage of productively and latently infected cells over total infection, we found that IL-15 stimulation minimally increased the percentage of productively infected cells (Fig. 5F) while decreasing the percentage of latently infected cells compared to IL-2 stimulation (Fig. 5G).

Since the increase in total infection in IL-15-stimulated cells was more pronounced with R5 HIV-GKO than X4/R5 HIV-GKO (Fig. 5D and Fig. 1C), we hypothesized that IL-15 might enhance the expression of CCR5 compared to IL-2. Indeed, flow cytometry analysis of CCR5 expression in CD4^+^ T cells stimulated with IL-15 or IL-2 revealed that IL-15 slightly increased both the percentage of CCR5^+^ cells as well as CCR5 MFI (Fig. 5H to J), suggesting that IL-15 may also favor HIV infection by increasing CCR5 expression in CD4^+^ T cells.

### T_SCM_ and T_CM_ cells express higher levels of CCR5 after IL-15 stimulation.

**FIG 6:**
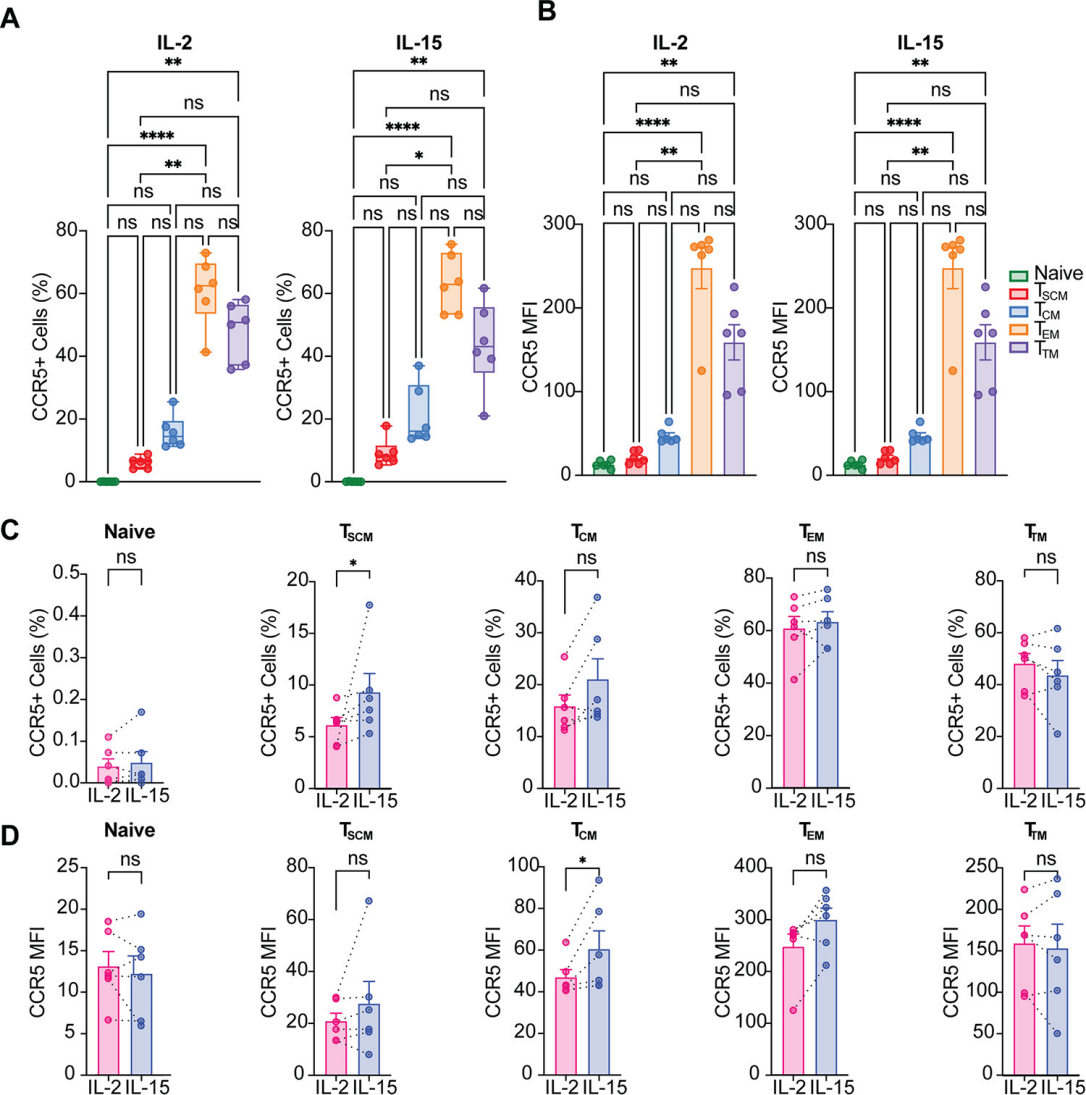
T_SCM_ and T_CM_ cells express higher levels of CCR5 after IL-15 stimulation. (A) Percentages of cells expressing CCR5 in each CD4^+^ T subset after IL-2 (left) or IL-15 stimulation (right) are shown. (B) Mean fluorescence intensity (MFI) of CCR5 for each subset after IL-2 (left) and IL-15 (right) stimulation. Significance was determined by Kruskal-Wallis test. ns, *P* > 0.05; *, *P* ≤ 0.05; **, *P* ≤ 0.01; ***, *P* ≤ 0.001; ****, *P* < 0.0001. (C) Bar graphs showing changes in CCR5 expression in each subset in the indicated conditions. (D) Graphs showing changes in CCR5 MFI in in the indicated conditions. Significance was determined by Wilcoxon signed-rank test. *, *P* ≤ 0.05; **, *P* ≤ 0.01; ***, *P* ≤ 0.001.

We next investigated how IL-15 influenced CCR5 expression in the different subsets with respect to IL-2. First, we determined expression in the different CD4^+^ T cells subsets after IL-2 or IL-15 stimulation. In both conditions, T_EM_ cells were the cells that showed higher expression of CCR5 in terms of percentage of positive cells as well as CCR5 MFI, followed by T_TM_, T_CM_, and T_SCM_ cells (Fig. 6A and B). As expected, naive T cells were virtually negative for CCR5 (Fig. 6A and B).

IL-15 stimulation slightly increased the percentage of T_SCM_ and T_CM_ cells expressing CCR5 and its levels of expression compared to IL-2 (Fig. 6C and D), suggesting that the overall increase in CCR5 levels observed in the total CD4^+^ T cells population (Fig. 5I and J) was mainly due to changes in CCR5 expression in T_CM_ cells.

### Levels of latency establishment after R5 HIV-GKO infection are comparable in T_CM_, T_TM_, and T_EM_ cells.

**FIG 7:**
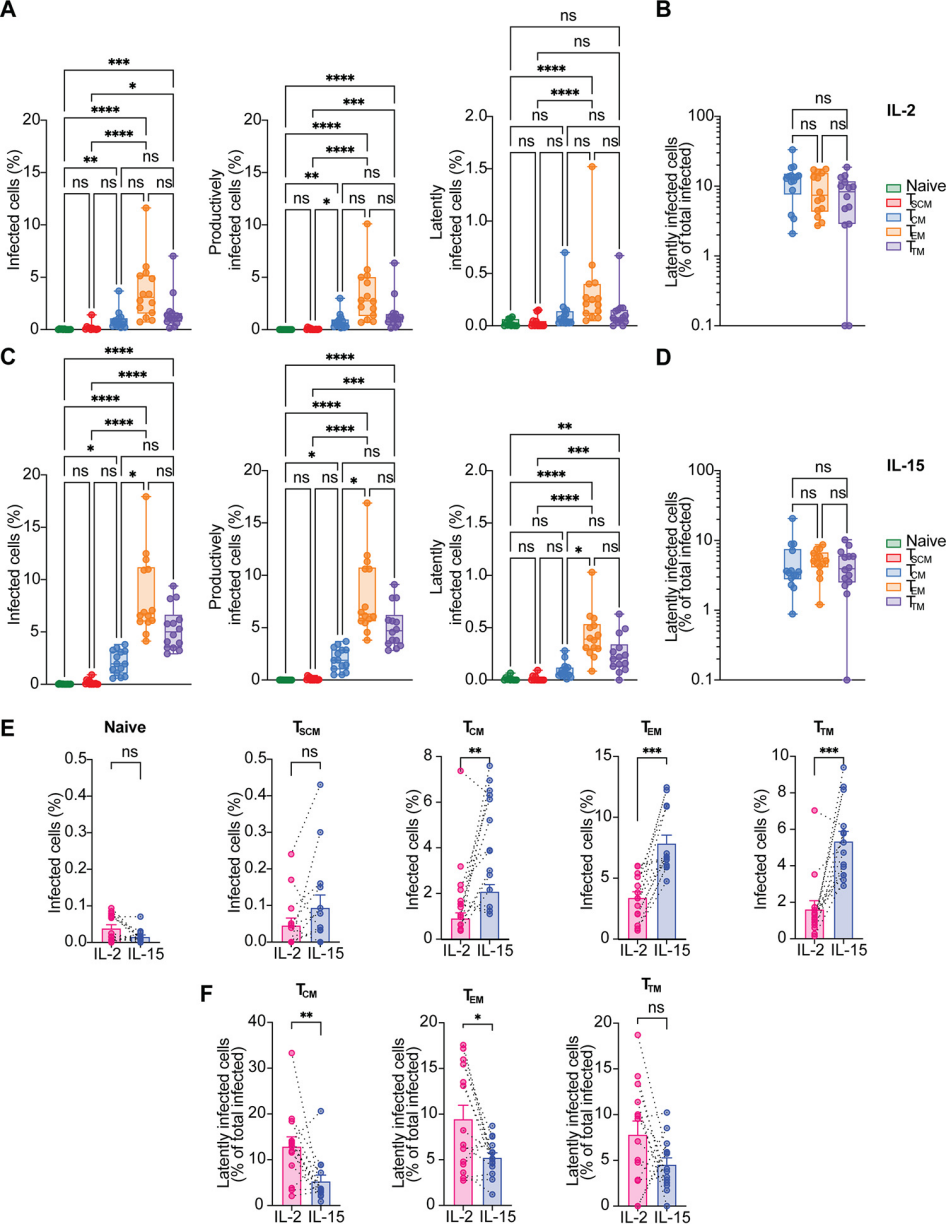
Levels of latency establishment after R5 HIV-GKO infection are comparable in T_CM_, T_TM_, and T_EM_ cells. (A) Box plots showing the percentage of total infected cells (left), productively infected cells (middle), and latently infected cells (right) in the different CD4^+^ T cell subsets after IL-2 stimulation with R5-HIV-GKO. (B) Percentage of latently infected cells with respect to total infection in each CD4^+^ T cell subset in IL-2 condition is shown. (C) Box plots showing the percentage of infected cells (left), productively infected cells (middle), and latently infected cells (right) in the CD4^+^ T cell subsets in IL-15 condition with R5-HIV-GKO. (D) Box plot showing the percentage of latently infected cells with respect to total infection in each CD4^+^ T cell subset after IL-15 stimulation. Significance was determined by Kruskal-Wallis test. ns, *P* > 0.05; *, *P* ≤ 0.05; **, *P* ≤ 0.01; ***, *P* ≤ 0.001; ****, *P* < 0.0001. (E) Bar graphs show changes in total infection with R5-HIV-GKO in the indicated conditions in the different CD4^+^ T cell subsets. (F) Percentage of latently infected cells with respect to total infection, in IL-2 or IL-15 stimulation, for each subset is shown. Significance was determined by Wilcoxon signed-rank test. *, *P* ≤ 0.05; **, *P* ≤ 0.01; ***, *P* ≤ 0.001.

We then sought to determine latency establishment in the different CD4^+^ T cell subsets in the context of the CCR5 bearing GKO. We stimulated CD4^+^ T lymphocytes for 3 days with IL-2 or IL-15, infected them with R5 HIV-GKO, and analyzed their immunophenotype by flow cytometry. As for the R5/X4 HIV-GKO, the highest number of infected cells was observed in the T_EM_ subset in both conditions. However, the difference with the other subsets was more striking and followed the expression of CCR5 (Fig. 7A and C, left panels). The distribution of productively and latently infected cells mirrored the total infection distribution (Fig. 7A and C, middle and right panels). The percentage of latently infected cells with respect to total infection was similar in all the more differentiated memory subsets (T_CM_, T_EM_, and T_TM_) (Fig. 7B and D). Naive and T_SCM_ cells expressed very low levels of CCR5 and were highly resistant to R5 HIV-GKO infection, and it was not possible to determine the percentage of latently infected cells with enough confidence. For this reason, they were left out of the analysis (Fig. 7B and D). IL-15 stimulation increased the overall infection for all the more differentiated memory subsets (T_CM_, T_EM_, and T_TM_). We observed a slight increase in T_SCM_ infection that did not reach statistical significance. As expected, the infection of naive T cells with R5 HIV-GKO was barely detectable (Fig. 7E). On the other hand, the percentage of latently infected cells with respect to total infection was diminished by IL-15 stimulation in all the memory subsets analyzed (T_CM_, T_EM_, and T_TM_) (Fig. 7F).

Taken together, these data suggest that, in our experimental system, IL-15 had a positive effect on overall infection in all T_SCM_, T_CM_, T_EM_, and T_TM_ cells, but the susceptibility of naive cells was unchanged.

### Productively infected cells are enriched in the more differentiated CD4^+^ memory subsets.

**FIG 8:**
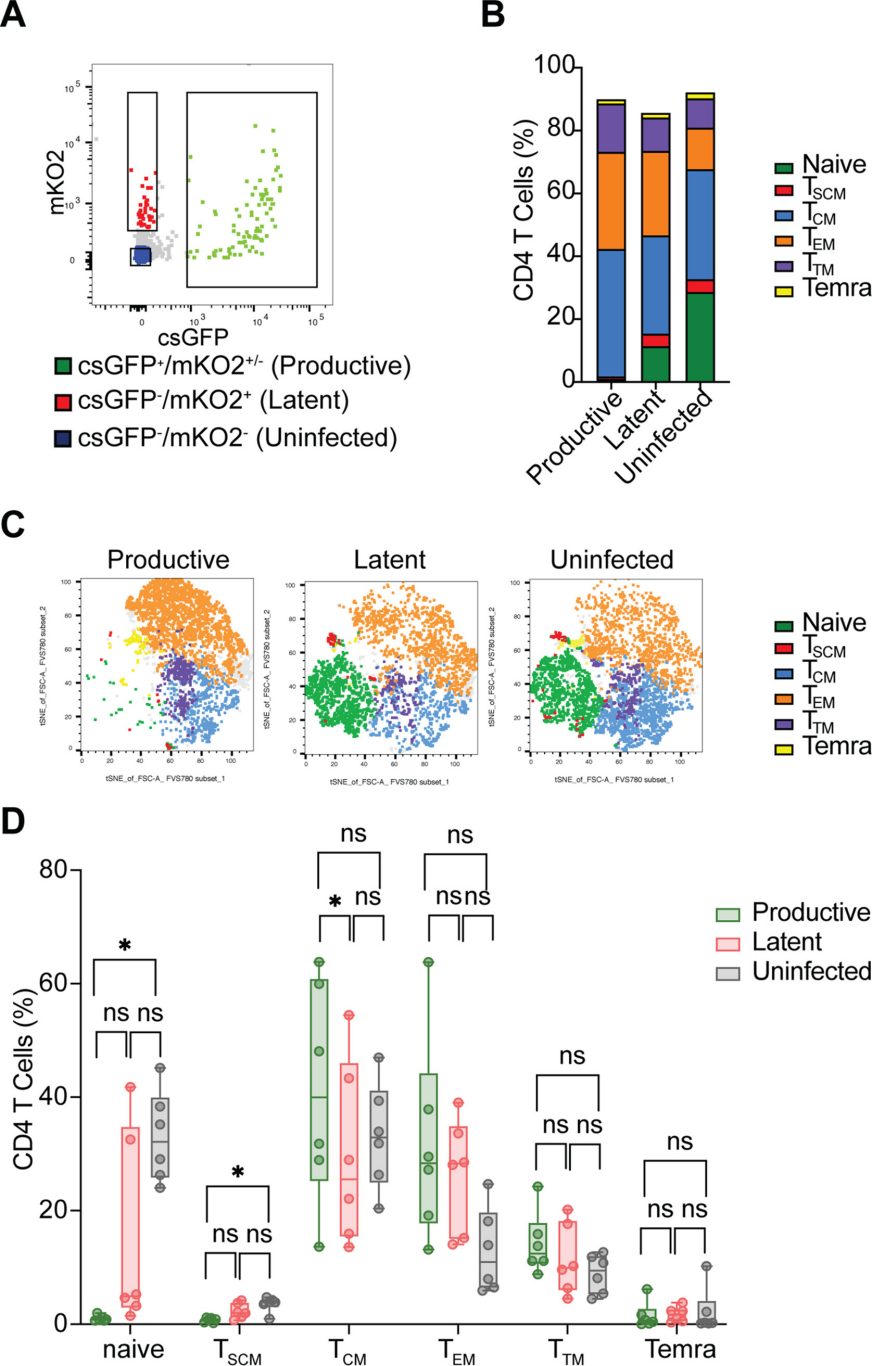
Productively infected cells are enriched in the more differentiated CD4^+^ memory subsets. (A) Gating strategy for the isolation of latently infected CD4^+^ T cells (mKO2^+^/csGFP^−^), productively infected cells (mKO2^+^/csGFP^+^ and mKO2^−^/csGFP^+^), and uninfected cells (mKO2^−^/csGFP^−^). (B) Stacked bar chart summarizing the percentage of the different CD4^+^ T cell subsets within uninfected, productively infected, and latently infected sorted cells (*n* = 6). (C) Representative t-SNE plots showing the global distribution of the different CD4^+^ T cell subsets within productively infected, latently infected, and uninfected sorted cells. (D) Percentage of each CD4^+^ T cell subset, productively infected (green bars), latently infected (red bars), and uninfected (gray bars) in infected sorted cells (*n* = 6). Significance was determined by Friedman test. ns, *P* > 0.05; *, *P* ≤ 0.05.

We next determined the cell composition of productively and latently infected cells as well as uninfected after stimulation with IL-15. To perform the analysis in a higher number of cells, we sorted latently infected cells (mKO2^+^/csGFP^−^), productively infected cells (mKO2^+^/csGFP^+^ and mKO2^−^/csGFP^+^) and uninfected cells (mKO2^−^/csGFP^−^) from six different donors (Fig. 8A) infected with dual-tropic enveloped HIV-GKO. The three cell populations were then stained with anti-CD4, anti-CD45RA, anti-CD45RO, anti-CD27, anti-CCR7, and anti-CD95 antibodies and subsequently analyzed by flow cytometry. We observed a marked decrease in the percentage of naive and T_SCM_ cells in productively infected cells compared to the uninfected population (Fig. 8B to D).

There was no significant difference in the T_SCM_ population between latently infected cells and uninfected, while the percentage of naive T cells in the latently infected population was very variable among donors. A slight increase (25%) in T_CM_ in productive cells compared to latent and uninfected was observed. The T_EM_ percentage was 2-fold higher in both the infected cell populations (productive and latent) than uninfected, and T_TM_ cells tend to be higher in the productive population, but these findings did not reach statistical significance. No differences in the Temra population were observed.

Overall, these data suggest that there is an underrepresentation of naive and T_SCM_ in productively infected cells. The general decrease in the percentage of naive was less pronounced in the latently infected cells and was minimal for T_SCM_ cells. On the other hand, the increase in the percentage of T_CM_ is more pronounced in the productively infected cells.

### CDK9 and cyclin T1 are equally expressed in the different CD4^+^ T cell subsets.

We have previously shown in this work that the higher levels of pTEFb expression upon IL-15 stimulation correlated with higher levels of productive infection than IL-2 (Fig. 1I, M, and N). We hypothesized that proclivity of naive and T_SCM_ cells to harbor a latent form of HIV compared to the other subsets may correlate with the expression of pTEFb complex. Expression of the core components of pTEFb is highly regulated in CD4^+^ T cells and macrophages, but how expression and activity are regulated in different CD4^+^ T cell subsets remains unclear (66–69).

**FIG 9:**
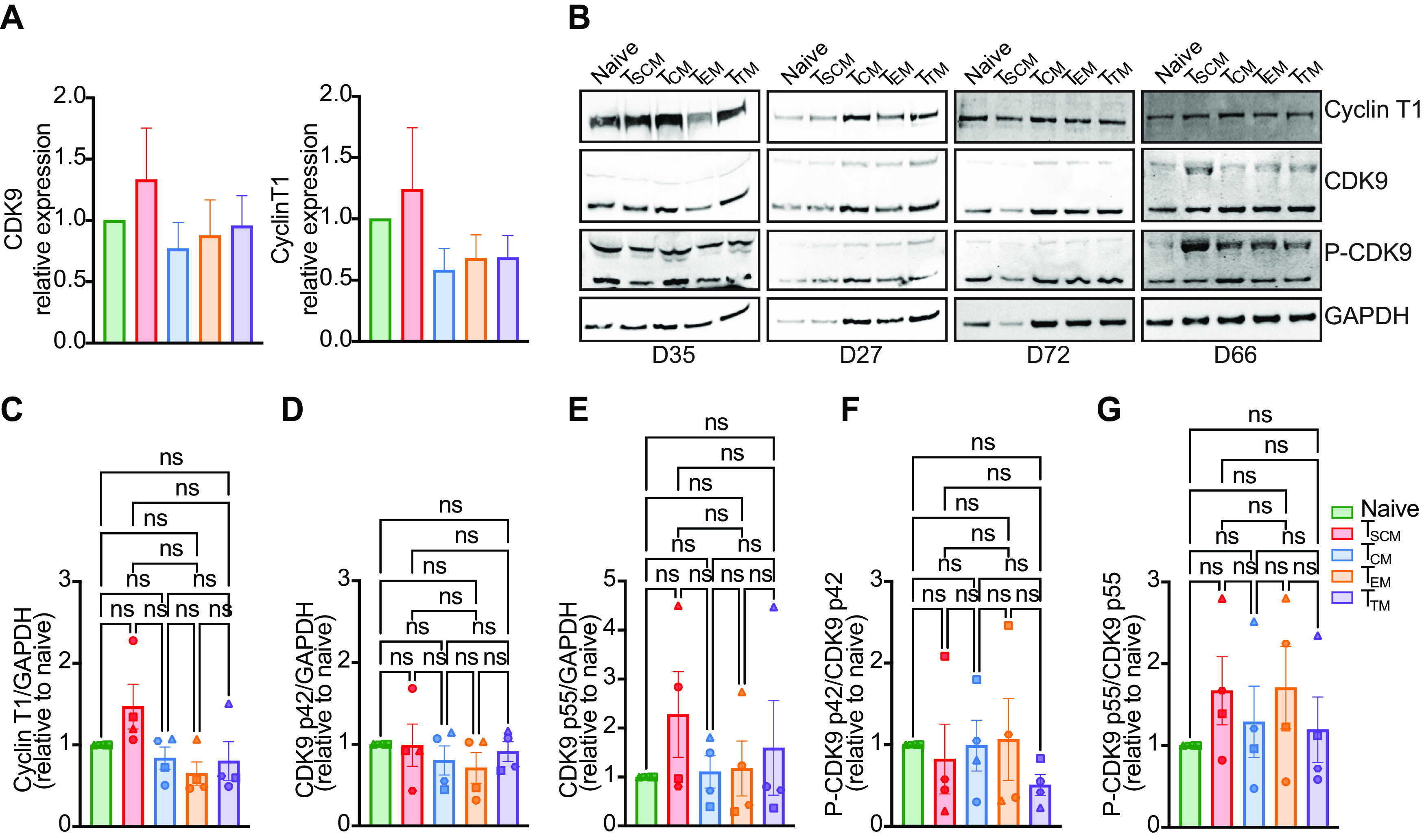
CDK9 and cyclin T1 are equally expressed in the different CD4^+^ T cell subsets. (A) Expression of CDK9 (left) and cyclin T1 (right) mRNA was analyzed in the different CD4^+^ T cell subsets. Expression in naive T cells was set as 1 (*n* = 2). (B) Cyclin T1 and CDK9 protein levels, as well as their phosphorylation status, were analyzed in cell lysates from sorted naive, T_SCM_, T_CM_, T_EM_, and T_TM_ cells. (C to G) Densitometric analyses of cyclin T1, CDK9 p42, CDK9 p55, P-CDK9 p42, and P-CDK9 p55, respectively. Expression levels in naive T cells were set as 1. Significance was determined by Kruskal-Wallis test. ns, *P* > 0.05; *, *P* ≤ 0.05; **, *P* ≤ 0.01; ***, *P* ≤ 0.001; ****, *P* < 0.0001.

We analyzed therefore the expression of CDK9 and cyclin T1 mRNA in the different CD4^+^ T cell subsets after 8 to 9 days of IL-15 stimulation, the specific time point at which CD4^+^ T cells were collected for the analysis of HIV infection. We sorted naive, T_SCM_, T_CM_, T_EM_, and T_TM_ cells from total CD4^+^ T lymphocytes. Analysis of mRNA isolated from the sorted subsets revealed that all the CD4^+^ T cell subsets express comparable levels of CDK9 and cyclin T1 mRNA (Fig. 9A).

Since no significant difference in mRNA expression was observed, we next investigated whether protein expression and CDK9 phosphorylation at residue threonine 186, known to be important in pTEFb activation and HIV transcription, were different in sorted naive, T_SCM_, T_CM_, T_EM_, and T_TM_ cells (70, 71). An equal number of sorted cells for each CD4^+^ T cell subset was analyzed for the expression of CDK9, phospho-CDK9, and cyclin T1 by Western blot analysis with GAPDH serving as a housekeeping control in four different donors (Fig. 9B). After densitometric analysis, no significant differences in cyclin T1 and CDK9 protein expression were observed among the CD4^+^ T cell subsets, and levels of phosphorylated CDK9 in position 186 were comparable among all the samples (Fig. 9C to G).

In conclusion, neither expression of pTEFb nor phosphorylation of CDK9 at residue threonine 186 seem to modulate the establishment of latency in the different T cell subsets.

### Analysis of NF-κB localization in the different CD4^+^ T cell subsets.

NF-κB, together with nuclear factor of activated T cells (NFAT), plays a pivotal role in HIV transcription (72, 73). NF-κB binds to two adjacent NF-κB/NFAT sites within the LTR promoter region and enhances the transcription of viral genes (72, 73). It has been previously shown that resting CD4^+^ T lymphocytes do not support a productive viral transcription, partially because of the unavailability of NF-κB in the nucleus (74, 75).

**FIG 10:**
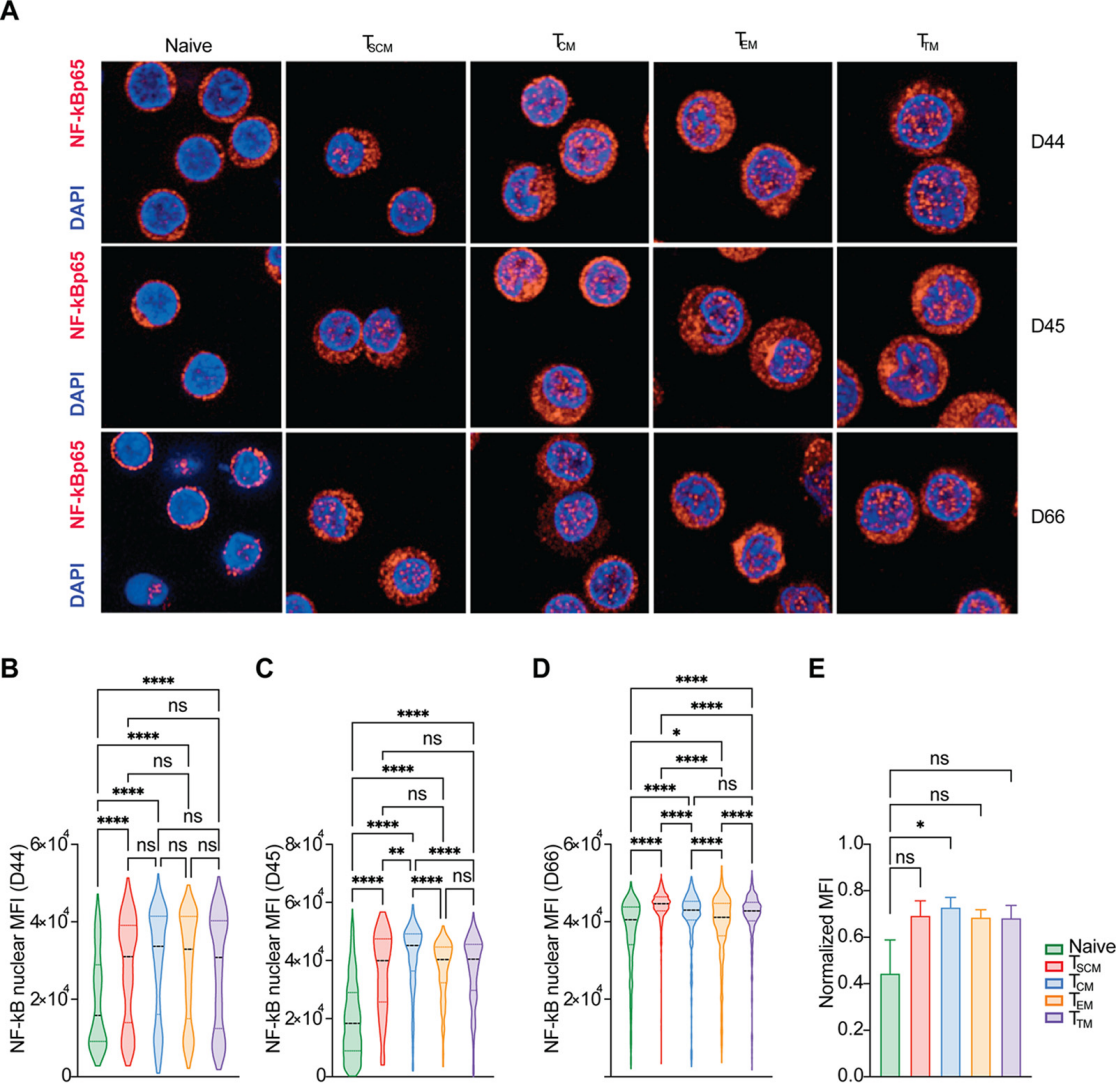
Analysis of NF-κB localization in the different CD4^+^ T cell subsets. (A) Confocal microscopy images of the different CD4^+^ T cell subsets, sorted from three different healthy donors, cultured for 8 to 9 days in IL-15. CD4^+^ T cell subsets cells were stained with anti-NF-κB antibody, a secondary antibody conjugated with Alexa 647 fluorophore and DAPI to visualize the nucleus (scale bars, 10 μm). (B to D) Mean fluorescence intensity of nuclear NF-κB-Alexa647 was quantified on a total of 1,286 cells for D44, 21,22 cells for D45, and 2,998 cells for D66. (E) For better comparison among the nuclear NF-κB mean fluorescence of the T cell subsets, all data from the three donors were normalized, and the median was calculated. Significance was determined by Kruskal-Wallis test. ns, *P* > 0.05; *, *P* ≤ 0.05; **, *P* ≤ 0.01; ***, *P* ≤ 0.001; ****, *P* < 0.0001.

Moreover, activation levels of the NF-κB pathway may be different in the CD4^+^ T cell subsets; in the absence of stimulation, T_CM_ cells have been found to have the highest levels of phosphorylated NF-κB in position 529, an indicator of NF-κB nuclear translocation, compared to T_TM_ and T_EM_ (43). We thus hypothesized that inefficient transcription of HIV provirus in naive and T_SCM_ cells might be caused by lower levels of nuclear NF-κB than the more differentiated CD4^+^ T memory cells. To test this hypothesis, we analyzed NF-κB cellular distribution in sorted CD4^+^ T cell subsets from three healthy blood donors following 8 to 9 days in culture with IL-15 (Fig. 10A). The nuclear MFI of NF-κB, calculated for each single identified cell object (mean, *n* = 427 analyzed cells per T cell subset, and mean, *n* = 1,820 cells for each donor), was lower in naive T cells than in all the other subsets in all three tested donors to different extents (Fig. 10B to D).

Nuclear NF-κB MFI was similar in all the memory subsets with interdonor variation. In two donors out of three, levels of nuclear NF-κB were slightly higher in T_CM_ than T_EM_ and T_TM_ cells despite showing similar levels of latency (Fig. 10C and D). To better compare the specific nuclear levels of NF-κB in the three donors among the different T cell subsets, we normalized the nuclear NF-κB MFI and calculated the median nuclear MFI for each subset. We observed that the median nuclear MFI of naive T cells was lower than the other memory subsets (Fig. 10E).

In conclusion, we observed that memory T cell subsets stimulated with IL-15 have a higher nuclear amount of NF-κB than naive CD4^+^ T cells. Most importantly, these data suggest that T_SCM_ cells are more similar to the other memory subsets than naive with respect to nuclear levels of NF-κB.

## DISCUSSION

In this study, we investigated HIV latency establishment in different primary human CD4^+^ T cell subsets in the presence of IL-15. This cytokine has been shown to be upregulated during acute infection, a time when the latent reservoir is established (4, 47, 48). Subsequent studies revealed that IL-15 increases the susceptibility of CD4^+^ T cells to HIV infection, in part by inducing phosphorylation and the consequent inactivation of the restriction factor SAMHD1 (35, 58, 59). On the other hand, very recently, the IL-15 superagonist N-803, in combination with CD8^+^ T cell depletion, has been proposed as a new tool to reactivate HIV from latency, both in *ex vivo* studies and in animal models, proving its role in modulating HIV transcription as well (46). However, no reduction of total viral DNA was observed, suggesting that activation of the IL-15 pathway was only partially efficient in reactivating latent provirus. Taking advantage of a dual fluorescent virus, which allows the discrimination of latently and productively infected cells, combined with immunostaining to determine the phenotype of the different CD4^+^ T cells in the peripheral blood, we investigated how latency is established in the different CD4^+^ T cells subsets cultured with IL-15 (49). In line with previous reports, we observed that when CD4^+^ T cells are stimulated with IL-15, the global levels of infection increase compared to IL-2 treatment. In addition, the percentage of productively infected cells, as measured by the LTR-driven expression of csGFP, was also higher with IL-15. Indeed, the levels of cyclin T1 and CDK9, proteins that play a pivotal role in HIV transcription, were increased in CD4^+^ T cells cultured in IL-15 compared to equimolar amounts of IL-2, suggesting that one of the mechanisms by which IL-15 increases HIV transcription is linked to the higher expression of the components of pTEFb complex (51, 53).

Immunophenotyping experiments revealed that naive CD4^+^ T cells and T_SCM_ cells were less susceptible to HIV infection, as previously reported (22, 31, 60). However, those two subsets were more prone to bear a latent form of the provirus compared to T_CM_, T_TM_, and T_EM_ cells once infected. In general, the observed higher rates of HIV transcription in differentiated CD4^+^ T cells subsets are in line with previous reports showing that highly differentiated T cells are more responsive to latency reversal (36, 37, 41). The latent reservoir is established in the early phases of infection by CCR5-tropic viruses; for this reason, we investigated latency establishment using a CCR5-pseudotyped HIV-GKO (4, 63–65). We confirmed that latency establishment was similar among T_CM_, T_EM_, and T_TM_ subsets, as observed for the R5/X4 HIV-GKO.

Unfortunately, we were not able to detect a sufficient level of infection of naive and T_SCM_ cells that could allow us to determine latency establishment in these subsets with the CCR5-tropic virus. The low susceptibility of these subsets to CCR5-tropic virus infection is due to the low expression of CCR5 on the plasma membrane (34). However, the presence of provirus in these subsets has been observed in both animal models and HIV^+^ individuals, suggesting that *in vivo* naive T cells and T_SCM_ are part of the latent reservoir (44, 76–79). Indeed, studies on animal models indicate that the contribution of naive T cells in the formation of the reservoir is particularly important in pediatric cases of HIV infection (78, 79). It is then important to also study latency establishment in naive T cells and T_SCM_ even though they are less susceptible to HIV infection compared to the more differentiated memory subsets.

We also investigated the molecular mechanism behind the increased tendency of naive CD4^+^ T cells and T_SCM_ to bear latent provirus. We observed that IL-15 stimulation compared to IL-2 increases both CDK9 and cyclin T1, the core components of the pTEFb complex. This is in line with previous reports indicating that in resting CD4^+^ T cells, CDK9 is expressed at low levels, and it is upregulated upon activation (80, 81). Considering the importance of pTEFb for HIV transcription, we hypothesized that naive CD4^+^ T cells and T_SCM_ cells express lower levels of its components. However, no difference in CDK9 and cyclin T1 mRNA and protein levels were observed. Analysis of the levels of phosphorylated CDK9 in position threonine 186, which has been shown to increase its activity, revealed that all the CD4^+^ T cell subsets analyzed contained comparable levels of P-CDK9 in position 186 in the IL-15 condition (70, 71). These data are in agreement with previous work showing that phosphorylation in position threonine 186 increases upon anti-CD3/anti-CD28 stimulation in both naive and memory CD4^+^ T cells to the same extent (82). Other posttranslational modifications of CDK9, such as acetylation in positions 44 and 48 and phosphorylation in position 175, have been implied to play a role in its activity (43, 83–85). Indeed, levels of P-CDK9 in position 175 have been shown to be very low in naive T cells, while among the memory subsets, T_TM_ cells express a higher level of this posttranslational modification of CDK9 (43). Further studies will be needed to determine whether these modifications play a role.

Finally, we investigated whether differences in NF-κB p65, another important factor for HIV transcription, were responsible for the different levels of latency establishment observed in the CD4^+^ T cell subsets considered for this study. Using confocal microscopy, we quantified the levels of total and nuclear NF-κB p65 in sorted naive, T_SCM_, T_CM_, T_TM_, and T_EM_ cells from three different donors cultured in IL-15 for 8 to 9 days. Overall, differences were modest, but naive T cells showed consistently lower levels of nuclear NF-κB p65, partially explaining the lower susceptibility of naive T cells to HIV transcription. These data suggest that the availability of transcription factors could be a limiting factor for HIV transcription in naive T cells rather than defects in Tat transactivation, even after IL-15 stimulation (74, 86, 87). Differences in nuclear NF-κB p65 in naive CD4^+^ T cells compared to memory subsets may reflect their lower responsiveness to IL-15 (62). Subcellular localization of NF-κB p65 in T_SCM_ cells was more comparable to the T_CM_, T_TM_, and T_EM_ cells than to naive CD4^+^ T cells, suggesting that the low permissivity of T_SCM_ to HIV transcription may be governed by other mechanisms rather than NF-κB p65 nuclear levels. Interdonor variation was observed in the nuclear levels of NF-κB p65 in the memory subsets. Despite showing similar levels of latency, T_CM_ contained higher levels of nuclear NF-κB p65 than T_EM_ and T_TM_, in line with what has been previously observed for phospho-NF-κB (43). This difference could be caused by differences in NF-κB kinetics: T_EM_ and T_TM_ could be more rapid in activating the negative feedback loop of the NF-κB pathway (88).

Recently, it has been shown that activation of the noncanonical NF-κB through second mitochondrion-derived activator of caspases (SMAC) mimetics induces reactivation of HIV latent provirus both *in vitro* and *in vivo*, but regulation of the noncanonical NF-κB pathway in the different CD4^+^ T cells is unknown (89–91).

Investigating whether the different CD4^+^ T cell subsets display similar sensitivity to SMAC mimetics would be an important step to maximize SMAC mimetics' effect on latency reversal.

Moreover, NFAT plays a pivotal role in HIV transcription and is involved in CD4^+^ T cell activation and differentiation. It has been shown that naive T cells contain very low levels of NFAT, but how NFAT is regulated in T_SCM_ cells is still unclear and deserves further detailed studies (92).

Another mechanism of latency establishment is epigenetic silencing and nuclear architecture surrounding integration sites (93–96). Epigenetic changes are critical components in T cell activation and differentiation processes. Previous works suggested that a chromatin-based mechanism limits cytokine responsiveness in cells that have not encountered their specific antigen. Specifically, nuclei of naive CD4^+^ T cells appeared characterized by condensed chromatin, which dispersed in response to T cell receptor (TCR) activation, but not to IL-2 treatment alone (97). T_SCM_ cells have already encountered the antigen but maintain robust replicative capacity and are multipotent. Studies in CD8^+^ T cells showed that the profile of H3K27me3 (associated with gene repression) and H3K4me3 (associated with gene expression in T cells) correlates in naive CD4^+^ T cells and T_SCM_ cells and it is segregated from T_CM_ and T_EM_ cells, suggesting that the epigenetic profile of T_SCM_ cells is more similar to naive rather than memory cells, thus explaining their correlation in latency rates versus productive infection rates (98).

This study has several limitations. First, the use of the dual-fluorescent virus could underestimate the frequency of latent infection in resting CD4^+^ T cells *in vitro* due to the poor constitutive expression of EF1α promoter in resting CD4^+^ T cells (99, 100). Second, we are considering only CD4^+^ T cells present in the peripheral blood, but other subsets of T cells present in lymphoid organs or tissues (like follicular T helper cells and Th17) are highly susceptible to infection and play a fundamental role in latency (101–104). In addition, we are not investigating the interaction between monocytes and DCs with different T cells subsets during infection. Indeed, monocytes and DCs are important in modulating HIV infection of resting T cells and latency establishment (105). Finally, we determined CDK9 and cyclin T1 expression, as well as NF-κB localization, at the time when we analyzed the outcome of infection; we cannot exclude that earlier time points might be relevant as well.

Our study, exclusively performed in primary human CD4^+^ T cells, analyzes not only latency establishment in different CD4^+^ T cell subsets but also pTEFb and NF-κB subcellular localization. We show differences in HIV latency establishment in different CD4^+^ T cell subsets from peripheral blood under IL-15 stimulation, a pathway able to induce HIV transcription in the absence of CD8^+^ T cells (46). Specifically, naive and T_SCM_ cells were more prone to latency than the other memory subsets. Interestingly, T_SCM_ cells were more similar to naive CD4^+^ T cells in the context of latency rates but more similar to T_CM_, T_TM_, and T_EM_ cells in the context of NF-κB levels and nuclear localization, suggesting that other mechanisms are responsible for the poor expression of HIV in these cells in the context of IL-15 stimulation. Given the importance of this small but long-lived subset in the biology of the latent reservoir and the disease progression, further studies are needed to dissect the molecular mechanism responsible for latency establishment in this specific CD4^+^ T subset (22, 23, 55, 56).

## MATERIALS AND METHODS

### CD4^+^ isolation and culture.

Buffy coats were obtained from anonymous healthy donors from Centro Trasfusionale, Ospedale Maggiore Policlinico Milano. Human peripheral blood mononuclear cells (PBMCs) were purified through density gradient centrifugation (Ficoll-Paque Plus; GE Healthcare), and CD4^+^ T cells were subjected to negative immunomagnetic isolation (CD4^+^ T cell isolation kit; Miltenyi Biotec) according to the manufacturer’s instructions. Purified CD4^+^ T cells were maintained in culture in RPMI 1640 (Euroclone) supplemented with 10% (vol/vol) heat-inactivated fetal bovine serum (FBS; Gibco), 100 U/mL penicillin (Euroclone), 0.1 mg/mL streptomycin (Euroclone), 1× minimal essential medium (MEM) nonessential amino acids (Gibco), 2 mM l-glutamine (Euroclone), 10 mM HEPES buffer solution (Gibco), and 1 mM sodium pyruvate (Gibco). Purified CD4^+^ T cells were stimulated with 20 ng/mL IL-15 (R&D Systems) or 20 UI/mL IL-2 (Miltenyi Biotec).

### Culturing of HEK 293TN.

Human embryonic kidney (293TN) cell line (System Bioscience; catalog no. LV900A-1) were cultured in Dulbecco’s modified Eagle medium (DMEM) (Euroclone) supplemented with 10% (vol/vol) heat-inactivated FBS (Gibco), 100 U/mL penicillin (Gibco), 0.1 mg/mL streptomycin (Sigma), 1× MEM nonessential amino acids (Sigma), and 2 mM l-glutamine (Sigma). Cells were maintained at 37°C in a 5% CO_2_ humidified incubator. Testing for mycoplasma was carried out using MycoAlert mycoplasma detection kit (Lonza).

### Plasmids.

The following reagents were obtained through the NIH-NIH AIDS Reagent Program, Division of AIDS, NIAID, NIH: HIV-1 92HT593.1 gp160 expression vector (pSVIII-92HT593.1) from Beatrice Hahn (catalog no. 3077) (57) and pSyngp140JR-FL. HIV Duo-Fluo II GKO LTR wild-type (WT) plasmid was a kind gift from Eric Verdin (49).

### Production of viral stocks.

HIV-GKO viral stocks were generated by calcium phosphate transient transfection of HEK 293TN cells with 10 μg of HIV-GKO plasmid and 5 μg of the R5/X4 envelope coding plasmid HIV-1 92HT593.1 or R5 envelope coding plasmid pSyngp140JR-FL in each 10-mm petri dish. The cell culture medium was replaced 8 h posttransfection, and supernatants were collected at 24 and 48 h posttransfection. The viral supernatant was filtered through a polyethersulfone (PES) 0.45-μM membrane and then ultracentrifuged for 2 h (Beckman; swinging-rotor SW 32 Ti; 80,000 × *g*) (106). Pellets were resuspended in 1:100 of initial volume in phosphate-buffered saline (PBS), aliquoted, and stored at −80°C. Viral stock concentration was assessed by HIV combo antigen-antibody enzyme-linked immunosorbent assay (ELISA; Dia.pro Diagnostic BioProbes).

### Viral infection.

IL-2 (20 UI/mL) or IL-15 (20 ng/mL) stimulated CD4^+^ T cells were spin infected with R5/X4 or R5-HIV-GKO for 2 h at 800 × *g* and 32°C in the presence of 2 μg/mL polybrene (Sigma) and left in the incubator for additional 2 h. The medium was then replaced with fresh complete RPMI supplemented with IL-2 or IL-15. We generally used 300 ng of p24 to infect 10^6^ CD4^+^ T cells as previously described (49, 106).

### Flow cytometry and cell sorting.

For flow cytometry analysis, between 2 × 10^6^ and 3 × 10^6^ of infected CD4^+^ T cells were stained and analyzed on a Symphony fluorescence-activated cell sorter (FACS) machine (BD Biosciences) or Cytek Aurora machine. Viability dye fixable viability stain 780 (FVS780; BD Biosciences) was used to discriminate between live and dead cells. The following antibodies were used: APC-R700 anti-CD27 (BD Biosciences), BV421 anti-CD95 (BioLegend), BV711 anti-CCR7 (BD Biosciences), APC anti-CD45RA (BioLegend), BUV805 or BUV605 anti-CD45RO (BD Biosciences), BUV395 anti-CD4 (BD Biosciences), and PE anti-CD195 (BioLegend). Briefly, CD4^+^ T cells were washed in PBS and incubated for 10 min in dark at room temperature (RT) with FVS780. Cells were then washed with magnetically activated cell sorting (MACS) buffer (Miltenyi Biotech) and incubated with the antibody mix in MACS buffer at 37°C for 20 min. Cells were washed and analyzed on Symphony FACS machine or Cytek Aurora. An average of 10^6^ cells were acquired per sample, and data were analyzed using FlowJo software (FlowJo, LLC).

For cell sorting of latently and productively infected and uninfected cells, between 20 × 10^6^ and 30 × 10^6^ of infected CD4^+^ T cells were filtered using 50-μm filters (Filcons, Syntec International) and sorted on cell sorter BD FACSAria III (BD Biosciences). Sorted cells were left in the incubator to recover for 2 h and then stained as described above. Between 0.4 × 10^5^ and 1 × 10^6^ of sorted CD4^+^ T cells were acquired on a Symphony FACS machine. For cell sorting of CD4^+^ T cell subsets from healthy donors for protein expression and RNA analysis, we stained between 30 × 10^6^ and 50 × 10^6^ CD4^+^ T cells with the following antibodies: APC-R700 anti-CD27 (BD Biosciences), BV421 anti-CD95 (BioLegend), BV711 anti-CCR7 (BD Biosciences), APC anti-CD45RA (BioLegend), PE anti-CD45RO (BioLegend), and APC/Fire750 anti-CD4 (BioLegend). Cells were stained at 37°C for 20 min, washed with MACS buffer, filtered, and sorted on Cell Sorter BD FACSAria III.

### Immunoblotting.

CD4^+^ T cells subsets were lysed in radioimmunoprecipitation assay (RIPA) buffer supplemented with complete protease inhibitor (Roche), sodium fluoride (10 mM), and sodium orthovanadate (1 mM), both from Sigma. Cell lysates were loaded into 4 to 12% SDS-PAGE gels (Bolt Bis-Tris precast gel; Thermo Fisher) and run at constant voltage (200 V) for 45 min. SDS-PAGE gels were transferred to a 0.2-μm nitrocellulose membrane (Amersham Protran Western blotting membrane; GE Healthcare) at constant voltage (90 V) for 1 h at 4°C. After transfer, membranes were blocked in 5% milk in 1× Tris-buffered saline with 0.1% Tween 20 (TBS-T) for 1 h with gentle rocking. After washing three times with TBS-T, membranes were incubated at 4°C overnight with the appropriate primary antibody and diluted 1:1,000 in bovine serum albumin (BSA) and 5% TBS-T. After secondary staining (secondary antibody diluted 1:5,000 in 5% milk in TBS-T), membranes were washed three times in TBS-T and developed. Sufficient ECL reagent (Amersham; GE Healthcare) was used to fully cover the membranes. Images were captured on an IBright FL 1500 instrument (Thermo Fisher). The following antibodies were used for detection: anti-SAMHD1 (Cell Signaling Technology; catalog no. 12361), Anti-phospho-SAMHD1 (Cell Signaling Technology; catalog no. 89930), anti-CDK9 (Cell Signaling Technology; catalog no. 2316), anti-phospho-CDK9 (Cell Signaling Technology; catalog no. 2549), anti-cyclin T1 (Cell Signaling Technology; catalog no. 81464), and anti-GAPDH (Cell Signaling Technology; catalog no. 2118).

### RNA isolation and quantitative real-time PCR.

Cellular RNA was isolated using PureLink RNA minikit (Invitrogen). RNA was treated with PureLink DNase (Invitrogen). cDNA was generated using the SuperScript Vilo cDNA synthesis kit (Thermo Fisher Scientific).

Gene expression was measured by quantitative real-time PCR using SensiFAST SYBR Lo-ROX kit (Bioline) according to the manufacturer’s instructions in a QuantStudio 5 real-time PCR system (Applied Biosystems). RSP11 was used as a housekeeping gene to normalize the data. The following primers were used: RSP11 forward, GCCGAGACTATCTGCACTAC; RSP11 reverse, ATGTCCAGCCTCAGAACTTC; CDK9 forward, CAAGTTCACGCTGTCTGAGA; CDK9 reverse, TAGCAGCCTTCATGTCCCTA; cyclinT1 forward, AACCTTCGCCGCTGCCTTC; and cyclinT1 reverse, ACCGTTTGTTGTTGTTCTTCCTCTC.

### Immunofluorescence.

CD4^+^ T cells subsets were sorted on Cell Sorter BD FACSAria III (BD Biosciences) using the following antibodies: BV421 anti-CD27 (BioLegend), PE anti-CD95 (BioLegend), PeCy7 anti-CCR7 (BioLegend), APC anti-CD45RA (BioLegend), BV605 anti-CD45RO (BD Biosciences), APC/Fire750 anti-CD4 (BioLegend), and green fluorescent Live/Dead fixable dead cell stain (Thermo Fisher). Naive, T_SCM_, T_CM_ T_EM_, and T_TM_ cells were cultured in RPMI complete supplemented with 20 ng/mL of IL-15 for 8 days in a 96-well U-bottom plate. For each CD4^+^ T cell subset, 80 × 10^4^ cells were seeded in duplicate on 96-well cell imaging plates (Eppendorf cell imaging plates) that were previously coated with poly-l-lysine 0.5 mg/mL overnight at 4°C. Cells were allowed to attach to the plate for 30 min at 37°C. Cells were then fixed with 4% paraformaldehyde (PFA) on ice for 15 min, washed 3 times in PBS, and then permeabilized with 0.3% Triton X-100 for 15 min. Next, cells were blocked in PBS, 5% fetal calf serum (FCS), 3% BSA, 2% goat serum, and 0.3% Triton X-100 for 1 h. Staining for NF-κB p65 was performed at 4°C overnight using anti-NF-κB p65 (Abcam; catalog no. ab32356; 1:100). After 5 washes with PBS, cells were incubated with a secondary antibody (goat anti-rabbit Alexa Fluor 647; Thermo Fisher) for 2 h. After 5 washes with PBS, nuclear staining was performed using DAPI (4′,6-diamidino-2-phenylindole; 1:500 in PBS). Isotype control was performed using goat IgG (Thermo Fisher) at the same concentration as the primary antibody. Cells were washed 3 times with PBS and then kept in 10% glycerol until analysis. Fluorescent images were captured with Leica SP5 confocal microscope (Leica Microsystems) and acquired using Las-AF v5 software.

### Confocal microscopy and digital imaging analysis.

CD4^+^ T cell populations from *n* = 3 donors seeded and immunolabeled for NF-κB plus DAPI nuclear labeling on 96-well optical plates (Eppendorf cell imaging plates) were semiautomatically acquired using a motorized stage mounted on an inverted true confocal laser scanning microscope (SP; Leica Microsystems) with 4 photomultiplier tube (PMT) detectors and 8 laser lines. Staining for NF-κB conjugated with Alexa-Fluor 647 labeling and DAPI nuclear staining were acquired simultaneously over two spectrally separated detectors, using 405 nm and 633 nm laser lines, through a 1-arbitrary unit (AU) pinhole aperture. A 63× oil objective (numerical aperture [NA], 1.43) was used coupled with a 3× zooming-in magnification. The best focal plan detection was achieved via an auto-focusing system, and the best Z plan was acquired for each field of view (FOV). At least *n* = 15 FOVs were acquired for each well, with a minimum of *n* = 8 cells centered in each FOV over at least *n* = 4 independent biological replicates for each T cell subset from every donor. Following acquisitions using the instrument software LAS-AF, images were processed via ImageJ for file conversion and then via NIS-Elements v.5.30 (Nikon-Lim Instruments) for advanced image processing and analysis. *Ad hoc*-designed pipelines of commands were set up for semiautomatic image processing and quantification. The Richardson-Lucy deconvolution algorithm was applied for a better appreciation of image details. Quantifications were performed via thresholding, binarization, and object segmentation on the GA3 module of NIS-Elements v.5.30. Binary masks for both DAPI signal and NF-κB-647 signal were designed cell based using the global threshold algorithm via pixel classification plus morphological size restriction factors. Combined masks for whole-cell segmentation were developed via Boolean operations and growing algorithm from DAPI-derived nuclei objects to water shedding of 640 signals. Secondary objects segmentations (cytoplasmic and nuclear NF-κB-enriched compartments) were calculated via binary subtraction. NF-κB compartmentalization in nucleus and cytoplasm, and over the whole cell, was then calculated for each single identified cell object, both as signal intensities (MFI), number of compartmentalized NF-κB protein objects, and signal or object ratios. Single-cell object-derived data were further elaborated via Excel and GraphPad software for plotting and statistics. Normalization was calculated by *X′* = (*X* − *X*_min_)/(*X*_max_ − *X*_min_) formula.
